# Clinical Application of a New Near-Infrared Light-Emitting Diode with Broader Spectrum for Skin Rejuvenation and Hair Growth Enhancement

**DOI:** 10.1007/s00266-025-05476-6

**Published:** 2025-12-09

**Authors:** Jong-Ho Kim, Junggyo Seo, Ayoung Koh, Minju Kim, Chan Yeong Heo

**Affiliations:** 1 Department of Plastic and Reconstructive Surgery, Seoul National University College of Medicine, Seoul National University Bundang Hospital, Seoul, Republic of Korea; 2 Korean Skin Research Center, Seongnam, Republic of Korea; 3 H&BIO Corporation/R&D Center, Seongnam, Republic of Korea; 4 Department of Biological Engineering, Graduate School of Konkuk University, Seoul, Republic of Korea

**Keywords:** Skin aging, Skin texture, Wrinkle, Skin elasticity, Skin moisture, Skin density, Hair count, New near-infrared light-emitting diode

## Abstract

**Background:**

Low-level light therapy (LLLT) and light-emitting diode (LED) therapy have gained popularity in aesthetic dermatology for their effectiveness and convenience in treating skin aging through photobiomodulation (PBM). Recent advancements have led to the development of a new near-infrared (nNIR) LED device with a broader spectrum, aiming to enhance skin rejuvenation and hair growth. This study aims to clinically evaluate the efficacy of this nNIR device.

**Methods:**

The study included two segments: skin rejuvenation and hair growth evaluation. For skin rejuvenation, 20 participants aged 45–59 used an nNIR LED facial mask for 30 minutes, five times a week over 12 weeks. Parameters such as wrinkle count, skin moisture, elasticity, and density were measured. For hair growth, 25 participants aged 20–55 applied the same device to the scalp daily for 20 weeks, assessing hair growth at 6, 13, and 20 weeks.

**Results:**

Skin rejuvenation showed significant improvements, with wrinkles decreasing by up to 27.22% and skin texture, elasticity, moisture, and density also improving. Hair growth evaluation indicated a 1.33% increase in hair count by 20 weeks, with high participant satisfaction and no adverse reactions reported.

**Conclusion:**

The nNIR LED device demonstrated clinical efficacy in skin rejuvenation and hair growth, highlighting the benefits of a broader spectral range in PBM. While further research with larger and more diverse populations is recommended, this nNIR LED device suggests significant potential for advancements in aesthetic treatments.

**Level of Evidence III:**

This journal requires that authors assign a level of evidence to each article. For a full description of these Evidence-Based Medicine ratings, please refer to the Table of Contents or the online Instructions to Authors www.springer.com/00266.

## Introduction

The skin is a vital organ, serving as a protective barrier against harmful environmental stimuli. Skin aging is an inevitable biological process characterized by the gradual loss of structural and functional characteristics [1, 2]. In the field of aesthetic dermatology, numerous approaches have been explored to address this condition, which include topical agents, light device, radiofrequency, injectable skin biostimulator, and surgical correction [2]. Among these methods, low-level light therapy (LLLT) and light-emitting diode (LED) therapy have emerged as major approaches and have been widely adopted due to their safety and convenience [3, 4]. These treatments work through photobiomodulation (PBM), where photons emitted by LEDs are absorbed by skin chromophores, resulting in changes in cellular activity, tissue regeneration, repair, and reduced inflammation [5, 6].

The National Aeronautics and Space Administration (NASA) first documented the therapeutic effects of near-infrared (NIR) light on skin in the 1990s, which subsequently led to the development of devices for skin rejuvenation using infrared radiation [7]. The NIR wavelength range spans from 780 to 2526 nm by the definition of the American Society for Testing and Materials. This NIR region is generally divided into two sub-regions: short wavelength NIR (780–1100 nm) and long wavelength NIR (1100–2526 nm) [8, 9]. Recent LLLT or PBM devices often utilize red and NIR wavelengths, with NIR proving effective for skin rejuvenation and hair growth in the cosmetic LED device market [10, 11]. Advancements in understanding the cascade of non-thermal cell signaling pathways in PBM have significantly increased the efficacy of devices in reducing wrinkles and improving skin texture [12]. Additionally, PBM has been confirmed to be effective for various skin conditions, including alopecia.[13]

Several studies have reported the existence of multiple action spectra that can induce PBM within the NIR range, suggesting the potential for increased efficacy with broader spectrum LLLT. [14, 15] To address these prospects, a new near-infrared (nNIR) LED has been developed, which features a broader spectrum than conventional narrow bandwidth NIR chips. This study aims to clinically evaluate the efficacy of this nNIR LED in skin rejuvenation and hair growth enhancement.

## Methods

### Subjects

The experiment consisted of two parts, evaluating both skin and hair growth. For skin evaluation, a total of 20 participants aged between 45 and 59 years, exhibiting eye wrinkles, rough and dry skin, and diminished skin elasticity, were enrolled. Exclusion criteria included individuals using medication known to influence study outcomes, those with skin disorders (e.g., photosensitivity, inflammation, rash, psoriasis, and skin cancer), skin allergies, or sensitivity in the test area, and pregnant or breastfeeding individuals.

**Fig. 1:**
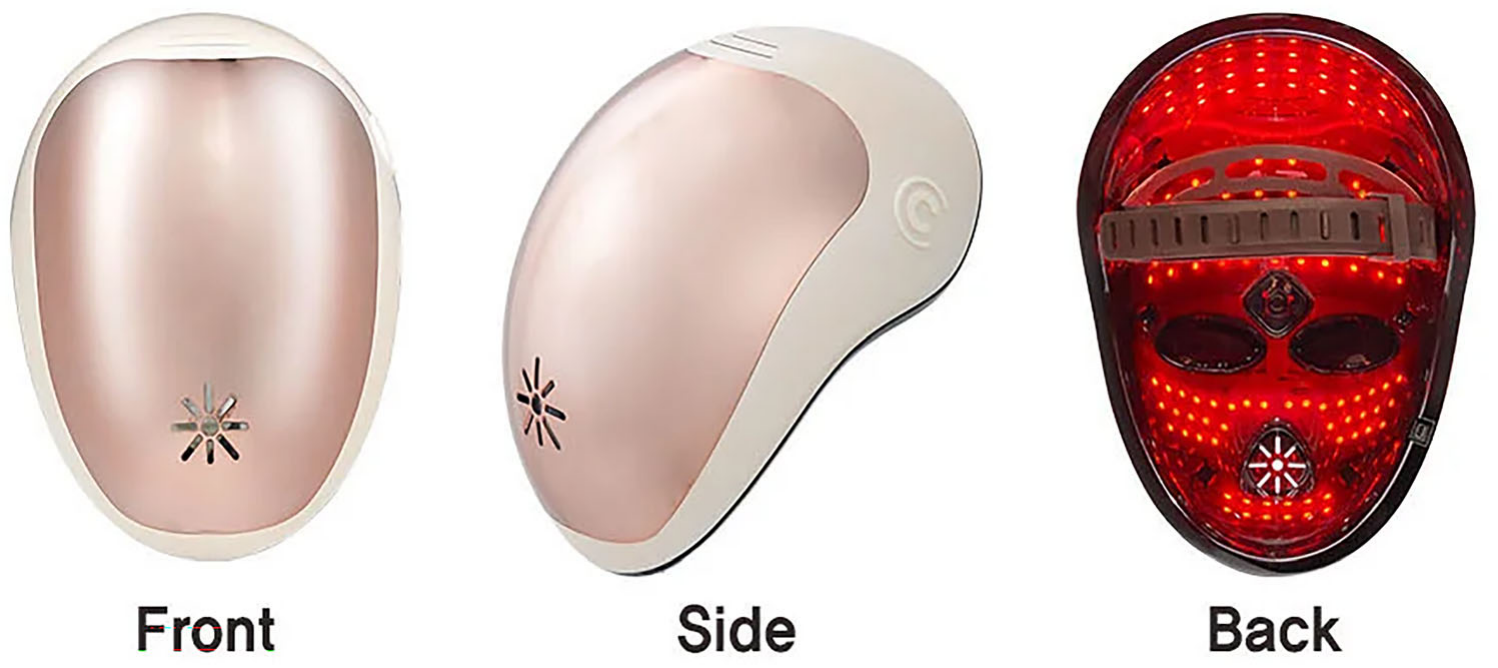
Photographs of the nNIR LED facial mask and scalp device used for skin rejuvenation and hair growth

The nNIR LED device (manufactured and provided by Samsung Electronics Co., Ltd.) used in this study is currently classified as a cosmetic beauty device under Korean regulatory standards. (Fig 1) Its design and testing procedures were conducted in accordance with institutional review board (IRB) approval (HBABN01-220801-HR-E0143-01)

To assess hair growth, 25 participants aged 20–55 years experiencing early-stage hair loss or expressing concerns regarding hair loss were recruited. Hair loss types were classified using the Basic and Specific (BASP) classification, and Ludwig criteria [16, 17]. After applying exclusion criteria, four individuals were excluded, resulting in a final analysis of 21 participants with a mean age of 37.81 ± 7.76 years. Exclusion criteria included individuals who had undergone hair transplant or scalp reduction surgery, those using medications known to affect hair growth, individuals with severe scalp conditions or infections, and those diagnosed with specific hair loss diseases such as alopecia areata, telogen effluvium, cicatricial alopecia, or androgenetic alopecia.

### Study Designs

**Fig. 2:**
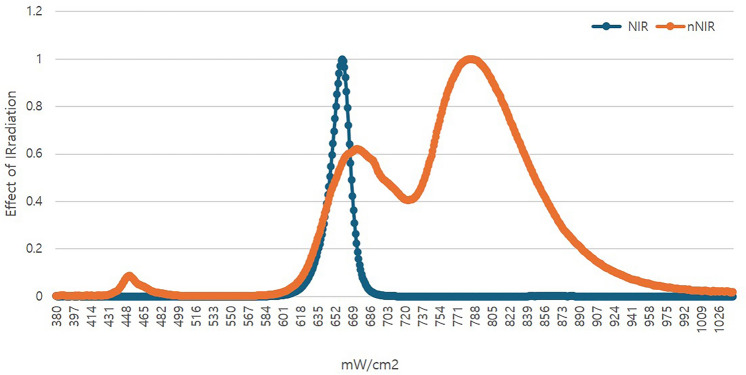
Broader spectrum of the nNIR with the spectrum profiles of conventional NIR

- Skin Rejuvenation
  The nNIR LED device, designed as a facial mask with an optical power of 0.81 mW/cm^2^, was applied to the face. The broader spectrum of the nNIR is illustrated alongside the spectrum profiles of various LEDs in Fig 2. Skin irradiation was administered five times weekly for 30 minutes, resulting in a cumulative dose of 87 J/cm^2^ over a 12-week period. Daily records were kept of the time before and after LED mask usage. Participants were instructed to apply the test product to their faces appropriately and leave it on for 30 minutes once daily, either in the morning or evening, after washing their faces. Measurements were conducted at four intervals: prior to product application, at 4 weeks, at 8 weeks, and at 12 weeks post-product use. These measurements included assessment of periorbital wrinkles, skin moisture levels, elasticity, and density. Skin roughness was also measured before and after 12 weeks of product use. Additionally, participants completed surveys, and researchers conducted observations and interviews to assess any adverse skin reactions.
- Hair Growth Evaluation
  The same device was utilized for hair growth evaluation, with the scalp receiving a cumulative dose of 146 J/cm^2^ over 20 weeks. Similarly, daily records were maintained of the time before and after LED mask usage for this evaluation.
  For hair growth evaluation, participants were instructed to cut their hair, and a 1 mm tattoo was applied to the selected area. The device was applied to the scalp and hair once daily for 30 minutes, either in the morning or evening. Hair growth evaluation was conducted at four intervals: prior to product application, at 6 weeks, at 13 weeks, and at 20 weeks post-product use. Adverse skin reactions were assessed at each time point through participant surveys, observations, and interviews with researchers.

### Objective Assessments

Eye wrinkles and skin texture were assessed using a 3-dimensional imaging system, PRIMOSCR (Canfield, USA), equipped with high-resolution sensors. Skin elasticity of the selected area was evaluated using a Cutometer® MPA580 (C+K, Germany). The device was applied to the skin three times for 2 seconds each, maintaining a constant negative pressure of 45 mbar, to quantify skin elasticity through graphical and numerical representations. Skin moisture levels were measured with the Corneometer® CM 825 (Epigem, Republic of Korea), which gauges water content across the epidermal layers by generating an electric field between the probe anode plates. Skin density was determined utilizing an Ultrasound probe (SNT Lab, Republic of Korea), which emitted sound pulses onto the skin, analyzed the resulting reactions, and depicted low density in dark hues and high density in bright hues based on signal intensities. Hair count assessments were conducted using a DSLR camera and Folliscope 5.0. Total hair count within a 1cm^2^ area was computed from the captured images (total hair count: number/cm^2^).

### Statistical Analysis

Statistical analyses were conducted using SPSS (version 22.0; SPSS Inc., Chicago, IL, USA). The normality of the data was assessed using the Shapiro–Wilk test, along with evaluations of kurtosis and skewness. For parametric comparisons of pre- and post-evaluation results, a paired t test was employed. In cases where data did not meet parametric assumptions, nonparametric tests including the Friedman test, Wilcoxon signed-rank test, and post hoc analysis with Bonferroni correction were used (*p* < 0.05). Homogeneity of variances between groups was checked using Levene’s test; groups were considered homogeneous if the p value exceeded 0.1. For comparisons between groups, if homogeneity of variances was confirmed, repeated-measures analysis of variance was used. If homogeneity of variances was not achieved, analysis of covariance was applied (*p* < 0.05).

## Results

### Skin Rejuvenation Assessment

**Fig. 3:**
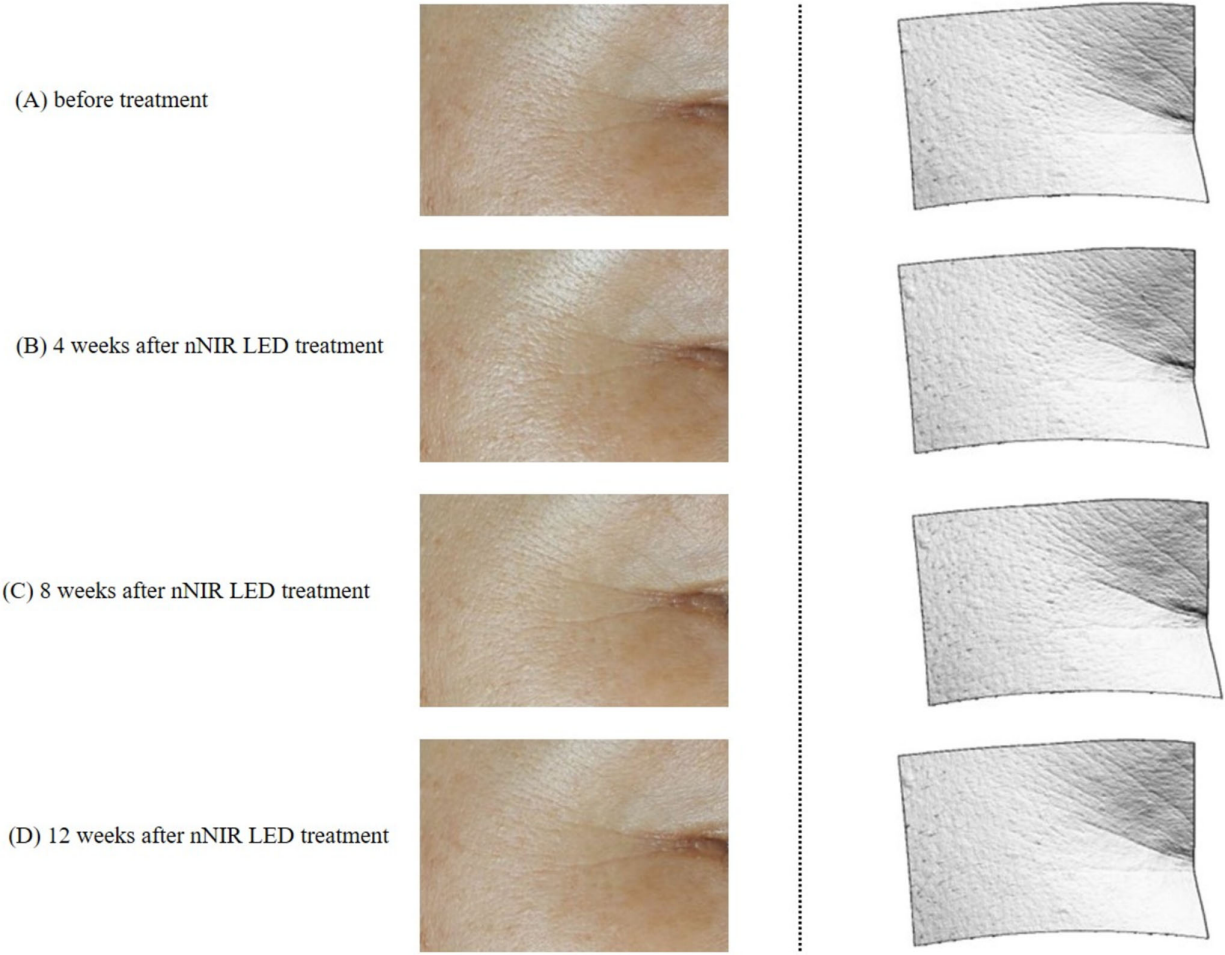
Improvement of eye wrinkles 4, 8, and 12 weeks after nNIR LED treatment. **A** before treatment; **B** 4 weeks after nNIR LED treatment; **C** 8 weeks after nNIR treatment; **D** 12 weeks after nNIR treatment

**Fig. 4:**
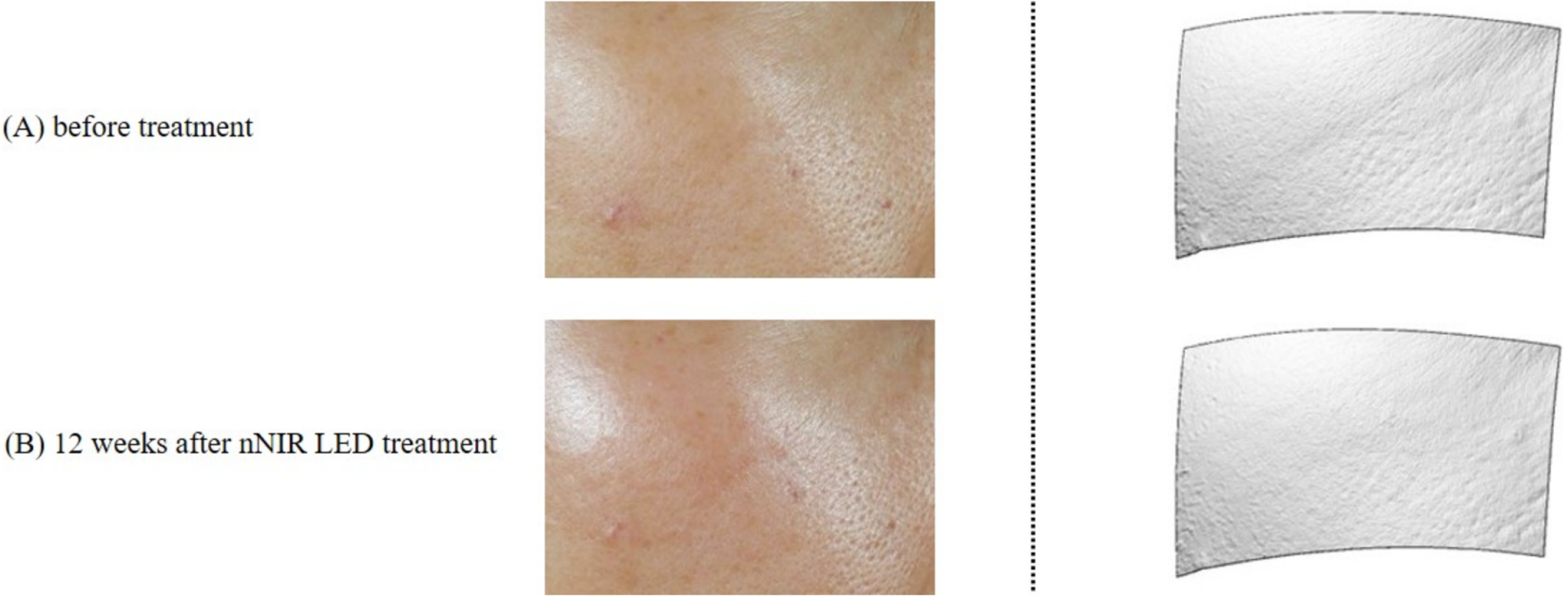
Improvement of skin texture 12 weeks after nNIR treatment; **A** before treatment; **B** 12 weeks after nNIR LED treatment

**Fig. 5:**
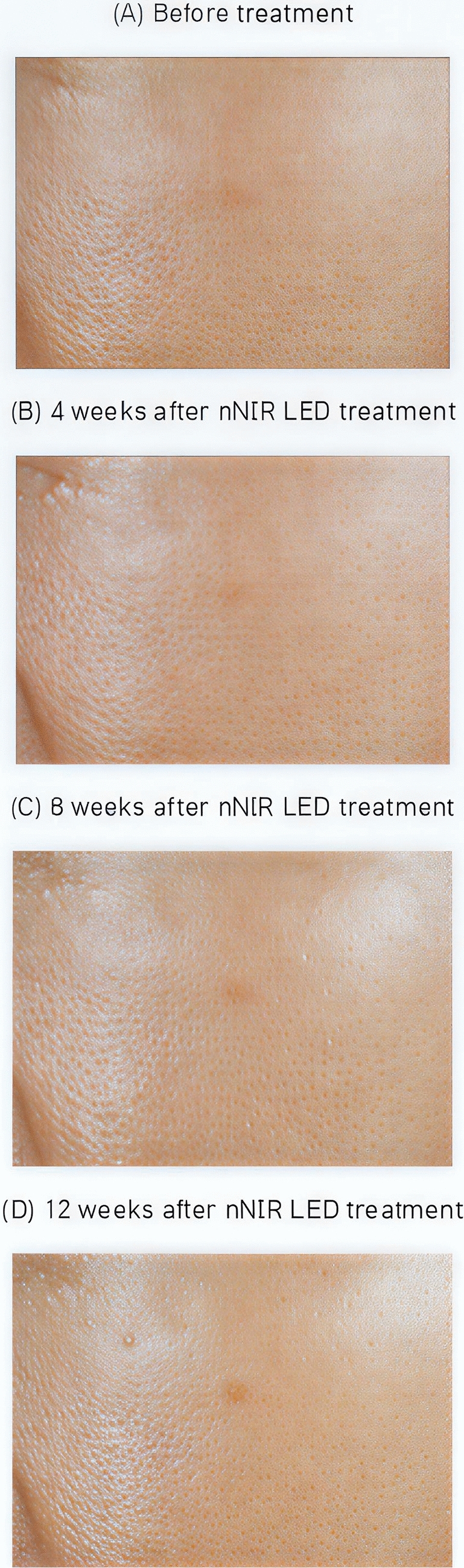
Improvement of skin moisture 12 weeks after nNIR LED treatment; **A** before treatment; **B** 4 weeks after nNIR LED treatment; **C** 8 weeks after nNIR LED treatment; **D** 12 weeks after nNIR LED treatment

**Fig. 6:**
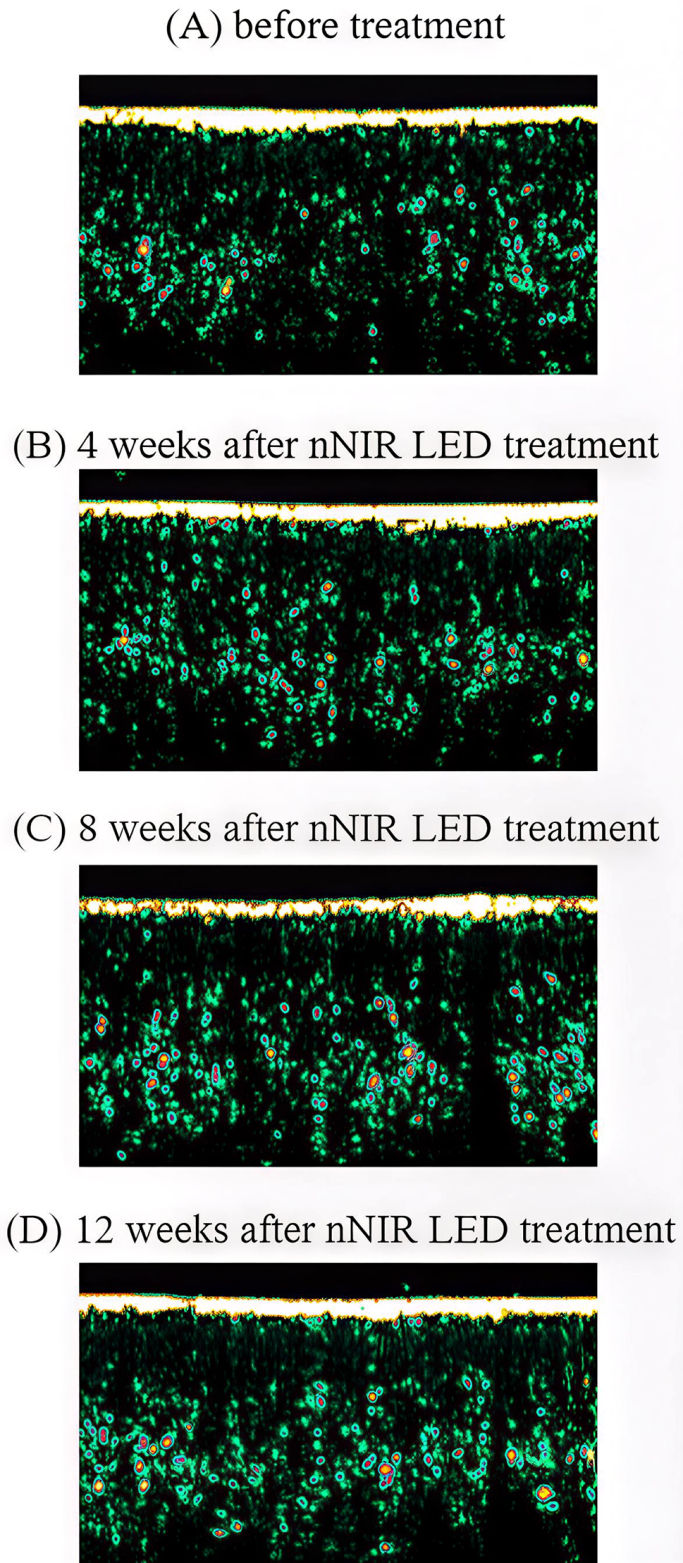
Improvement of skin density 12 weeks after nNIR LED treatment; **A** before treatment; **B** 4 weeks after nNIR LED treatment; **C** 8 weeks after nNIR LED treatment; **D** 12 weeks after nNIR LED treatment

A cohort of 20 participants (mean age 51.45 ± 4.12 years) was included. The treatment demonstrated significant improvements across various skin parameters: total wrinkle count reduced by 24.62% and 27.22% after 4 and 8 weeks, respectively, with a continued 26.48% decrease observed 12 weeks post-treatment (Fig 3). Additionally, skin texture exhibited a reduction in maximum roughness values by 5.13% after 12 weeks (Fig 4). Furthermore, significant enhancements were observed in skin elasticity, with an 8.66% increase noted 12 weeks post-treatment, alongside a noteworthy increase in skin moisture levels by 6.74% (Fig 5). Skin density also showed improvement, with a significant 3.60% increase after 12 weeks of treatment, as indicated by darker coloration in hybrid projection (Fig 6). A comprehensive summary of the measurement data is presented in Table 1.

**Table 1:** Statistical analysis of before and after treatment comparison results for skin rejuvenation by time point

Parameter	Time point	Mean $\pm$ SD	*P*-value	% Change
Average depth of wrinkles $(\mu m$)	Pre-treatment	55.25 $\pm$ 13.85		
	4 weeks	55.70 $\pm$ 15.60	0.748	$+$ 0.81
	8 weeks	55.95 $\pm$ 13.57	0.585	$+$ 1.27
	12 weeks	52.10 $\pm$ 15.27	0.029^*^	$-$ 5.70
Maximum depth of biggest wrinkle $(\mu m$)	Pre-treatment	286.70 $\pm$ 147.01		
	4 weeks	278.90 $\pm$ 148.25	0.619	$-$ 2.72
	8 weeks	276.30 $\pm$ 161.45	0.542	$-$ 3.63
	12 weeks	255.40 $\pm$ 143.61	0.037^*^	$-$ 10.92
Total wrinkle count (number)	Pre-treatment	142.75 $\pm$ 50.77		
	4 weeks	107.60 $\pm$ 42.02	0.000^*^	$-$ 24.62
	8 weeks	103.90 $\pm$ 38.60	0.000^*^	$-$ 27.22
	12 weeks	104.95 $\pm$ 39.88	0.000^*^	$-$ 26.48
Average roughness $(\mu m$)	Pre-treatment	27.79 $\pm$ 6.78		
	4 weeks	30.04 $\pm$ 6.92	0.179	$+$ 8.10
	8 weeks	29.87 $\pm$ 6.71	0.390	$+$ 7.48
	12 weeks	28.31 $\pm$ 7.25	0.422	$+$ 1.87
Average maximum height of the profile $(\mu m$)	Pre-treatment	372.28 $\pm$ 83.78		
	4 weeks	385.12 $\pm$ 73.26	0.156	$+$ 3.45
	8 weeks	382.64 $\pm$ 73.09	0.502	$+$ 2.78
	12 weeks	357.15 $\pm$ 75.33	0.179	$-$ 4.06
Average roughness $(\mu m$) Maximum roughness depth $(\mu m$)	Pre-treatment	16.16 $\pm$ 3.68		
	12 weeks	15.25 $\pm$ 3.02	0.049^*^	$-$ 5.63
	Pre-treatment	124.65 $\pm$ 28.33		
	12 weeks	118.26 $\pm$ 24.33	0.043^*^	$-$ 5.13
Skin elasticity (%)	Pre-treatment	63.17 $\pm$ 6.60		
	4 weeks	64.81 $\pm$ 4.85	0.275	$+$ 2.60
	8 weeks	66.34 $\pm$ 5.86	0.049^*^	$+$ 5.02
	12 weeks	68.64 $\pm$ 6.40	0.001^*^	$+$ 8.66
Skin moisture (A.U.)	Pre-treatment	61.10 $\pm$ 9.77		
	4 weeks	61.78 $\pm$ 10.09	0.143	$+$ 1.11
	8 weeks	63.03 $\pm$ 9.78	0.010^*^	$+$ 3.16
	12 weeks	65.22 $\pm$ 7.88	0.000^*^	$+$ 6.74
Skin density (%)	Pre-treatment	13.90 $\pm$ 1.41		
	4 weeks	14.03 $\pm$ 1.35	0.097	$+$ 0.94
	8 weeks	14.33 $\pm$ 1.30	0.000^*^	$+$ 3.09
	12 weeks	14.40 $\pm$ 1.20	0.000^*^	$+$ 3.60

^*^P<0.05 was considered significant

### Hair Growth Evaluation

A cohort of 21 participants (mean age 37.81 ± 7.76 years) was included, consisting of 11 individuals with BASP-M1F1 and 10 with Ludwig type 1. The analysis revealed increases in hair growth values of 0.52% after 6 weeks and 1.33% after 20 weeks of treatment, respectively. The improvement observed at 20 weeks was statistically significant. A detailed summary of the measurements is provided in Table 2. Survey results on the effects and usability of the product indicated positive responses from 71.4% of participants at 6 weeks, 81.0% at 13 weeks, and 85.7% at 20 weeks. No adverse skin events were reported among any of the study participants during the study period.

**Table 2:** Statistical analysis of before and after treatment comparison results for hair count by time point

Parameter	Time point	Mean $\pm$ SD	*P*-value	% Change
Total hair counts ($numbe{r/cm}^{2}$)	Pre-treatment	110.43 $\pm$ 21.58		
	6 weeks	111.00 $\pm$ 21.94	0.271	$+$ 0.52
	20 weeks	111.90 $\pm$ 21.39	0.003^*^	$+$ 1.33

^*^P<0.05 was considered significant.

## Discussion

The widespread adoption of LEDs has revolutionized the lighting source by replacing traditional fluorescent light. Unlike sunlight, which comprises approximately 6% ultraviolet, 47% visible light, and 53% NIR radiation, LEDs convert electrical energy into light and emit a narrow range of visible light, typically spanning from 400 nm to 700 nm. LEDs have gained substantial interest in dermatology from both dermatologists and estheticians, owing to their optimal properties for PBM and notable benefits, including ease of compliance, user-friendliness, straightforward application, and absence of pain [6, 18]. This device has also demonstrated significant efficacy in the treatment of wound healing and various skin conditions, including psoriasis, acne vulgaris, and actinic keratosis [19]. There have been some studies on the effects of LED therapy on skin rejuvenation and hair growth, particularly focusing on the effects of red or NIR light. Nam et al. evaluated the efficacy and safety of two types of LLLT using 660 nm red LEDs and 411–777 nm white LEDs on facial wrinkles in 52 women [20]. While both LED treatments significantly decreased the depth, maximum, and smoothness of wrinkles over 12 weeks, the red LEDs showed a more notable reduction in wrinkle volume. Russell et al. found that combination light therapy using 633 and 830 nm wavelengths significantly improved facial skin texture and reduced periorbital wrinkles in 81% of participants [21]. Lanzafame et al. conducted a double-blind randomized controlled trial involving 44 males with androgenetic alopecia, demonstrating that LLLT using a device emitting 655 nm light significantly increased hair counts, with a 39% increase in the active group compared to the placebo [13]. Subsequent studies involving female patients also reported that LLLT is effective for promoting hair growth.[22]

In this study, we confirmed clinical results indicating the efficacy of a newly developed nNIR device with a broader wavelength range compared to conventional LED devices in promoting skin anti-aging and hair growth restoration. This extends findings from animal experiments using a broader spectrum nNIR LED device [23]. The nNIR device emits a spectrum covering 600–900 nm, significantly broader than the 20–30 nm range of conventional narrow bandwidth NIR LED chips. Our study confirmed the effectiveness of LED sources, as validated by animal experiments, and provided clinical support for Karu’s action spectrum. [24] Karu’s research revealed that there is no single action spectrum for cellular responses relevant to phototherapy and showed that cytochrome c oxidase, located in the mitochondrial respiratory chain, is activated by red and near-infrared light [14, 15]. Therefore, the action spectrum for biological responses often spans multiple wavelengths, highlighting the advantage of using a broad-spectrum approach that covers a wider spectral range. Based on these findings, our study confirmed the clinical efficacy and safety of the newly developed nNIR device.

Compared with commercially available beauty devices that typically use narrow band LEDs (emission range of approximately 20–30 nm), the nNIR LED device used in this study emits over a broader wavelength range (600–900 nm), enabling simultaneous activation of multiple photobiomodulation pathways. This broader spectral coverage may lead to more effective stimulation of cytochrome c oxidase and enhanced tissue penetration, thereby improving skin rejuvenation and hair growth outcomes. These findings align with and extend previous reports by Nam et al., Russell et al., and Lanzafame et al., which demonstrated wavelength-dependent variability in LED efficacy. Thus, our nNIR device design offers a potential advantage in achieving superior clinical outcomes compared to conventional narrow band devices.

While this study validates the clinical efficacy of the nNIR device, it also has several limitations. Although statistically significant effects on skin rejuvenation and hair growth were observed, the small sample size necessitates further research with a larger cohort to validate these findings. Additionally, outcomes were assessed only up to 12 or 20 weeks post-treatment, highlighting the need for longer-term follow-up to fully evaluate the effects of the nNIR device. The study period covered different seasons, and environmental conditions such as temperature and humidity were not systematically recorded. Since all assessments were conducted at a single research center under standard indoor conditions, the variation was minimized but not completely excluded. Therefore, seasonal and environmental changes may still have influenced skin or hair parameters. Furthermore, the relatively homogenous patient population suggests that future trials should include a more diverse range of genders and age groups to better assess the device’s efficacy across different demographics. Moreover, comparing the nNIR device with existing NIR devices could enhance the study’s value and provide a more comprehensive evaluation of its relative performance.

In conclusion, the nNIR LED device demonstrated clinical efficacy in skin rejuvenation and hair growth, highlighting the benefits of a broader spectral range in PBM. Significant improvements were observed in skin texture, elasticity, moisture, and density, along with increased hair growth. While further research with larger and more diverse populations is recommended, these results support the potential of the nNIR LED device to advance aesthetic treatments.
